# Incidence, Etiologies, and Outcomes of Respiratory Distress in Newborns Admitted to the Neonatal Intensive Care Unit of a Tertiary Care Teaching Hospital in Rural South-West Bihar

**DOI:** 10.7759/cureus.112245

**Published:** 2026-07-07

**Authors:** Richa Raj, Neha Kumari, Om Prakash Singh, Mani Kant Kumar

**Affiliations:** 1 Pediatrics, Employees' State Insurance Corporation Medical College, Bihta Patna, Patna, IND; 2 Pediatrics, Katihar Medical College and Hospital, Katihar, IND; 3 Pediatrics, Narayan Medical College and Hospital, Sasaram, IND

**Keywords:** neonatal outcomes, neonatal respiratory distress, nicu admissions, respiratory distress syndrome, transient tachypnea of the newborn

## Abstract

Background

One of the most frequent reasons for neonatal intensive care unit (NICU) hospitalization is respiratory distress, which causes significant early neonatal morbidity and mortality, especially in resource-constrained environments. Etiology and related risk factors need to be identified early to enhance the outcome of neonates. The aim of the study was to establish the prevalence, etiology, and prognosis of respiratory distress in infants admitted to the NICU of a tertiary care teaching hospital in rural South-West Bihar.

Methods

This is a prospective observational study, which was carried out in the level III NICU of a tertiary care teaching hospital during 18 months (April 2021-September 2022). Neonates between 0 and 28 days of age who were hospitalized with clinical manifestations of respiratory distress were all included. Maternal and neonatal histories were documented in detail. The severity was determined using the Downe's score on term neonates and the Silverman-Anderson score on preterm neonates. Relevant radiological and laboratory inquiries were conducted to ascertain the etiology. Statistical Package for Social Sciences (SPSS) version 27 (IBM Corp., Armonk, NY) was used to analyze the data, and descriptive statistics was obtained.

Results

A total of 110 neonates with respiratory distress were enrolled. Male neonates constituted 60% of cases. The majority (62%) presented within the first 24 hours of life. Cesarean section accounted for 54.5% of deliveries, and 67.3% were inborn. Moderate respiratory distress was observed in 45.4% of neonates, while 31% had severe distress. Transient tachypnea of the newborn (TTN) was the most common etiology, followed by perinatal asphyxia and respiratory distress syndrome (RDS). Significant associations were observed between etiology and gestational age, birth weight, and need for resuscitation at birth. Overall, 82.7% of neonates were discharged successfully, while the mortality rate was 8.3%.

Conclusion

Respiratory distress remains a major cause of NICU admissions in rural tertiary care settings. Transient tachypnea of the newborn was the most common cause, with prematurity and low birth weight being significant risk factors. Early recognition, prompt management, and improved perinatal care can significantly enhance neonatal survival outcomes.

## Introduction

Respiratory distress (RD) is one of the most common causes of admission to the neonatal intensive care unit (NICU) among both term and preterm neonates. It encompasses a broad spectrum of clinical conditions, ranging from mild transient respiratory adaptation to severe respiratory failure requiring advanced ventilatory support. Clinically, respiratory distress is characterized by tachypnea, nasal flaring, intercostal and subcostal retractions, grunting, cyanosis, and decreased breath sounds, reflecting increased work of breathing and impaired oxygenation [1]. Early recognition and timely management are critical, as delayed intervention may lead to respiratory failure, hypoxemia, and cardiopulmonary arrest [2].

Respiratory morbidity contributes substantially to neonatal morbidity and mortality worldwide. Approximately 15% of term infants and nearly 29% of late preterm infants admitted to NICUs develop significant respiratory complications, with an even higher incidence among infants born before 34 weeks of gestation [3]. In India, the neonatal mortality rate has gradually declined in recent years owing to improvements in perinatal and neonatal care; however, respiratory disorders continue to be a major contributor to neonatal mortality [4].

The incidence of respiratory distress is inversely related to gestational age. Transient tachypnea of the newborn (TTN) and respiratory distress syndrome (RDS) are more frequently observed in preterm and late preterm infants [5]. Several maternal and perinatal factors increase the risk of neonatal respiratory illness, including prematurity, cesarean section delivery, meconium-stained amniotic fluid, gestational diabetes mellitus, pregnancy-induced hypertension (PIH), chorioamnionitis, oligohydramnios, and inadequate antenatal care [6]. Increasing rates of cesarean delivery have also been associated with rising respiratory morbidity among neonates.

The etiology of neonatal respiratory distress is diverse and includes transient tachypnea of the newborn, respiratory distress syndrome, meconium aspiration syndrome (MAS), perinatal asphyxia, neonatal sepsis, congenital heart disease, persistent pulmonary hypertension of the newborn, congenital diaphragmatic hernia, and air leak syndromes such as pneumothorax [7,8]. Although advances in neonatal ventilation strategies, continuous positive airway pressure (CPAP), surfactant replacement therapy, and improved monitoring have significantly improved survival rates, outcomes remain dependent on gestational age, birth weight, underlying etiology, and timely intervention [9].

Despite advances in neonatal care, data regarding the incidence, etiological spectrum, and outcomes of neonatal respiratory distress from rural tertiary care centers in India remain limited. Therefore, the present study was conducted to evaluate the clinical profile, etiological spectrum, and outcomes of neonates admitted with respiratory distress to the NICU of a tertiary care teaching hospital in rural South-West Bihar.

## Materials and methods

Study design and setting

This prospective observational study was conducted in the Level III Neonatal Intensive Care Unit (NICU) of the Department of Pediatrics at Narayan Medical College and Hospital, Jamuhar, Sasaram, a tertiary care teaching hospital located in rural South-West Bihar, India. The study was carried out over an 18-month period from April 2021 to September 2022.

Ethical approval

Ethical approval was obtained from the Institutional Ethics Committee of Narayan Medical College and Hospital prior to the commencement of the study (approval number: GNSU/RPEC/2020/46/20, date: 14/01/2021). Written informed consent was obtained from the parents or legal guardians of all enrolled neonates.

Study population

All neonates aged 0-28 days admitted to the NICU with clinical features suggestive of respiratory distress during the study period were considered eligible for inclusion. Respiratory distress was identified clinically by the presence of one or more of the following signs: tachypnea, nasal flaring, chest retractions, grunting, cyanosis, or decreased air entry. Consecutive universal sampling was employed, whereby all eligible neonates fulfilling the inclusion criteria during the study period were enrolled.

Inclusion and exclusion criteria

Neonates presenting with signs of respiratory distress within the first 28 days of life were included in the study. Neonates with major congenital anomalies, chromosomal abnormalities, or surgical conditions requiring urgent intervention were excluded.

Data collection

A detailed maternal and neonatal history was obtained at admission. Information regarding antenatal care, maternal illnesses, premature rupture of membranes, mode of delivery, intrapartum complications, and the need for resuscitation at birth was recorded.

Gestational age was estimated based on the first day of the last menstrual period. In cases of uncertain dates, findings from third-trimester ultrasonography were considered, and when both were unavailable, the New Ballard scoring system was used.

Birth weight and other anthropometric measurements were documented at admission. Demographic details, clinical features, laboratory findings, and outcomes were systematically recorded using a predesigned data collection proforma.

Assessment of severity

The severity of respiratory distress was assessed using the Downe's scoring system for term neonates and the Silverman-Anderson scoring system for preterm neonates, as described in previous studies [10].

Investigations and management

Relevant laboratory investigations and radiological evaluations were performed as clinically indicated to establish the etiological diagnosis. Management was provided according to standard NICU protocols. Neonates were followed up until discharge, death, referral, or leaving against medical advice (LAMA), and the outcomes were recorded.

Etiological diagnosis

Etiological diagnosis was established using predefined clinical, laboratory, and radiological criteria. Transient tachypnea of the newborn (TTN) was diagnosed in neonates presenting with respiratory distress shortly after birth, characteristic chest radiographic findings (prominent pulmonary vascular markings, fluid in interlobar fissures, or hyperinflation), and spontaneous resolution within 72 hours. Respiratory distress syndrome (RDS) was diagnosed primarily in preterm neonates with respiratory distress requiring oxygen support and chest radiographs demonstrating diffuse reticulogranular opacities with air bronchograms. Meconium aspiration syndrome (MAS) was diagnosed in neonates born through meconium-stained amniotic fluid who subsequently developed respiratory distress with compatible clinical and radiological findings. Perinatal asphyxia was diagnosed based on a history of perinatal insult, low Apgar scores, requirement of resuscitation at birth, and clinical evidence of hypoxic-ischemic encephalopathy. Congenital heart disease was confirmed by echocardiography. Congenital diaphragmatic hernia and pneumothorax were diagnosed based on clinical presentation and radiological findings.

Statistical analysis

Data were entered into Microsoft Excel (Microsoft Corp., Redmond, WA) and analyzed using Statistical Package for Social Sciences (SPSS) version 27 (IBM Corp., Armonk, NY). Descriptive statistics were used for data analysis. Continuous variables were summarized as mean and standard deviation, whereas categorical variables were presented as frequencies and percentages. The findings were summarized in tables and described narratively.

## Results

Baseline characteristics

During the 18-month study period, a total of 110 neonates with clinical features of respiratory distress were admitted to the NICU. Baseline demographic and perinatal characteristics are summarized in Table 1. Among the enrolled neonates, 66 (60.0%) were male and 44 (40.0%) were female. The majority of neonates presented within the first 24 hours of life (68, 61.8%), followed by presentation on day 2 (24, 21.8%) and day 3 (18, 16.4%). Regarding the mode of delivery, 60 (54.5%) neonates were delivered by lower segment cesarean section (LSCS), while 50 (45.5%) were delivered vaginally. Most neonates were inborn (74, 67.3%), whereas 36 (32.7%) were outborn.

**Table 1:** Baseline Characteristics of Neonates (N = 110) LSCS: lower segment cesarean section

Variable	Category	Number	Percentage (%)
Gender	Male	66	60.0
	Female	44	40.0
Day of presentation	Day 1	68	61.8
	Day 2	24	21.8
	Day 3	18	16.4
Mode of delivery	LSCS	60	54.5
	Vaginal	50	45.5
Place of birth	Inborn	74	67.3
	Outborn	36	32.7

Severity of respiratory distress

Severity assessment using Downe's score for term neonates and the Silverman-Anderson score for preterm neonates showed that moderate respiratory distress was the most common presentation, observed in 50 (45.5%) neonates, followed by severe distress in 34 (30.9%) and mild distress in 23 (20.9%) neonates (Table 2).

**Table 2:** Severity of Respiratory Distress

Severity	Number	Percentage (%)
Mild	23	20.9
Moderate	50	45.5
Severe	34	30.9

Maternal risk factors

Among the mothers, 57 (51.8%) had no identifiable comorbidity. Pregnancy-induced hypertension was the most common maternal risk factor, affecting 17 (15.5%) mothers, followed by anemia in 14 (12.7%), eclampsia in 10 (9.1%), gestational diabetes mellitus in 8 (7.3%), and hypothyroidism in 4 (3.6%) mothers (Table 3).

**Table 3:** Maternal Risk Factors

Maternal condition	Number	Percentage (%)
No comorbidity	57	51.8
Pregnancy-induced hypertension	17	15.5
Anemia	14	12.7
Eclampsia	10	9.1
Gestational diabetes mellitus	8	7.3
Hypothyroidism	4	3.6

Etiology of respiratory distress

Transient tachypnea of the newborn (TTN) was the most common etiology of respiratory distress, accounting for 42 (38.2%) cases. This was followed by perinatal asphyxia in 28 (25.5%) cases, respiratory distress syndrome (RDS) in 18 (16.4%) cases, and meconium aspiration syndrome (MAS) in 13 (11.8%) cases. Less frequent causes included congenital heart disease (7, 6.4%), congenital diaphragmatic hernia (1, 0.9%), and pneumothorax (1, 0.9%) (Table 4).

**Table 4:** Etiological Distribution of Respiratory Distress TTN: transient tachypnea of the newborn, RDS: respiratory distress syndrome, MAS: meconium aspiration syndrome

Etiology	Number	Percentage (%)
TTN	42	38.2
Perinatal asphyxia	28	25.5
RDS	18	16.4
MAS	13	11.8
Congenital heart disease	7	6.4
Congenital diaphragmatic hernia	1	0.9
Pneumothorax	1	0.9
Total	110	100.0

Clinical outcome

The majority of neonates were discharged after successful treatment (91, 82.7%). Mortality was observed in 9 (8.2%) neonates, while 7 (6.4%) left against medical advice (LAMA) and 3 (2.7%) were referred to higher centers for advanced management (Table 5).

**Table 5:** Clinical Outcome of Neonates LAMA: left against medical advice

Outcome	Number	Percentage (%)
Discharged	91	82.7
Death	9	8.2
LAMA	7	6.4
Referred	3	2.7

## Discussion

Respiratory distress is among the most common reasons for neonatal intensive care unit (NICU) hospitalization and one of the leading causes of early neonatal morbidity and mortality. In our current study at a teaching hospital with a tertiary care in rural South-West Bihar, respiratory distress was mostly noted in male babies, with an increased number of cases observed within the first 24 hours of the birth of the newborn. Similar male predominance has been reported; respiratory distress was more prevalent in the case of male newborns and may be attributed to the later development of the lungs and the effects of hormones on surfactant production [11]. Similar results state male preponderance in NICU admissions with respiratory causes [12].

The majority of neonates in our study presented within the first day of life, highlighting the importance of perinatal factors in the development of respiratory distress. Early-onset respiratory distress has been strongly associated with conditions such as transient tachypnea of the newborn (TTN), respiratory distress syndrome (RDS), and perinatal asphyxia [13]. It is reported that over 60% of neonates with respiratory distress manifested symptoms within the first 24 hours, consistent with our findings [14].

A higher proportion of affected neonates in our study were delivered by cesarean section. Previous studies have reported an association between cesarean delivery and neonatal respiratory morbidity, particularly transient tachypnea of the newborn, owing to delayed clearance of fetal lung fluid [15]. However, the present study did not differentiate between cesarean deliveries performed before or after the onset of labor; therefore, no conclusions regarding the influence of labor status can be drawn from our data.

In our cohort, transient tachypnea of the newborn emerged as the most common etiology, followed by perinatal asphyxia and respiratory distress syndrome. This etiological distribution aligns with findings from studies conducted in similar tertiary care settings in India, where TTN accounted for the majority of early neonatal respiratory distress cases [16]. Conversely, RDS remains more prevalent among preterm neonates due to surfactant deficiency and structural lung immaturity [17-19].

Maternal comorbidities such as pregnancy-induced hypertension (PIH), anemia, and diabetes mellitus were notable contributors in our population. Maternal hypertension has been linked to placental insufficiency and fetal hypoxia, which may predispose neonates to perinatal asphyxia and respiratory complications [20]. Additionally, poorly controlled maternal diabetes is known to delay surfactant synthesis, increasing the risk of RDS even in late preterm or term infants.

The overall survival rate in our study was favorable, with more than four-fifths of neonates discharged successfully. The mortality rate was 8.3%, which is comparable to findings from other tertiary care centers in developing regions [21]. Mortality in neonatal respiratory distress is often influenced by factors such as prematurity, severity of illness at presentation, delayed referral, and availability of advanced ventilatory support [22].

The study provides valuable insights into the epidemiological pattern of neonatal respiratory distress in a rural tertiary care setting. Rural healthcare systems often face challenges including delayed referrals, limited antenatal supervision, and inadequate perinatal care. Strengthening antenatal risk identification, promoting institutional deliveries, and ensuring availability of skilled neonatal resuscitation can potentially reduce the burden of respiratory morbidity [23].

This study has several limitations. First, as a single-center prospective observational study conducted at a rural tertiary care hospital, the findings may not be fully generalizable to other healthcare settings. Second, the observational design limits the ability to establish causal relationships between risk factors and outcomes, and residual confounding due to unmeasured maternal and neonatal variables cannot be completely excluded. Third, 7 (6.3%) neonates left against medical advice (LAMA), and 3 (2.7%) neonates were referred to higher centers; therefore, their outcomes could not be ascertained. This may have introduced attrition bias and could have resulted in the underestimation of the actual mortality rate. Finally, long-term neurodevelopmental follow-up was not performed, particularly among neonates with perinatal asphyxia and severe respiratory distress. Future multicenter studies with larger sample sizes and long-term follow-up are warranted to validate these findings and better identify predictors of adverse outcomes.

Despite these limitations, the study has several strengths. The prospective study design allowed systematic and standardized data collection, reducing the risk of missing information. Furthermore, the study provides important data on the incidence, etiological spectrum, and outcomes of neonatal respiratory distress from a rural tertiary care center in South-West Bihar, a region where published evidence remains limited. The findings contribute valuable information for improving neonatal care and resource planning in similar resource-constrained settings.

## Conclusions

Respiratory distress continues to be a leading contributor to neonatal morbidity and mortality, particularly in rural and resource-constrained settings. In this study, transient tachypnea of the newborn emerged as the most common cause, followed by perinatal asphyxia and respiratory distress syndrome. Most neonates presented within the first 24 hours of life, and moderate respiratory distress was the predominant clinical presentation.

Despite the burden of disease, the overall survival rate was encouraging, reflecting the impact of timely NICU intervention and standardized management protocols. Strengthening antenatal screening, promoting institutional deliveries, ensuring skilled birth attendance, and improving early neonatal resuscitation practices may further reduce morbidity and mortality associated with neonatal respiratory distress. Continued research and multicentric studies are recommended to refine preventive strategies and optimize neonatal outcomes in similar rural healthcare settings.
